# Attrition of health extension workers in Ethiopia: trends, regional variations and determinants – a mixed methods study of 15 years of experience

**DOI:** 10.1186/s12913-023-10292-2

**Published:** 2023-12-20

**Authors:** Habtamu Milkias Wolde, Merhawi Gebremedhin Tekle, Yibeltal Kiflie Alemayehu, Esie Gebrewahd Gebre, Alula M. Teklu

**Affiliations:** 1 Jimma University, Institute of Health, Jimma, Ethiopia; 2 Federal Ministry of Health, Addis Ababa, Ethiopia; 3 School of Public Health, Haramaya University, Harar, Ethiopia; 4 MERQ Consultancy PLC, Addis Ababa, Ethiopia; 5 School of Geography and Environmental Studies, Haramaya University, Harar, Ethiopia

**Keywords:** Attrition, Health extension workers, Regional variation, Trend, Ethiopia

## Abstract

**Background:**

Ensuring regular supervision, capacity building and motivation are crucial for the successful retention of health extension workers (HEWs). Failure in these aspects could increase the attrition rate of HEWs. To date, there has not been a comprehensive nationwide study on HEW attrition that could act as a source of evidence for policy makers. This study explored HEW attrition, including leaving the health sector entirely and its regional variation, trends and predictors of attrition out of the health system.

**Methods:**

This study explored the attrition of HEWs from the beginning of the program until the end of 2018. A district-based mixed method study was conducted to review the personnel files of HEWs. A multistage sampling technique was employed to select 3,476 HEWs, and a probability weight was assigned for each observation. Descriptive statistics were calculated for the outcome and predictor variables. A logistic regression model was used to model attrition out of the health system. A qualitative study was conducted to understand the reasons why HEWs leave their jobs. Thematic analysis was performed using Nvivo version 12.

**Results:**

The magnitude of attrition of HEWs was found to be 21.1% during the fifteen years of HEP implementation. Of the total 704 who left their job as an HEW, 530 (73%) left the health system altogether. Number of biological children [AOR = 0.61, 95% CI; 0.42–0.89], having an additional education [AOR = 8.34, 95% CI; 3.67–18.98], obtaining official recognition [AOR = 0.29, 95% CI; 0.10–0.83], administrative reprimand [AOR = 1.66, 95% CI; 1.07 -2. 56), distance between district health office and health post [AOR = 1.75, 95%CI; 1.18–2.59) and COC status [AOR = 2.06, 95%CI 1.39–3.06) were independent predictors of leaving the health sector. High regional variation in attrition was observed, ranging from 38.5% in Addis Ababa to just 6.1% in the Harari region. The trend of attrition has steadily increased over time, with a high of 1,999 attritions per 10,000 HEW in 2018. Psychosocial factors, administrative issues, career advancement incentives, and workplace-related problems were the themes that emerged from the qualitative study as reasons for attrition of HEWs.

**Conclusion:**

Even though the magnitude of attrition was relatively low, there was high regional variation and incremental trends. Moreover, the out-of-health sector attrition is also high. Critically examining the HEP policy environment to increase the number of HEWs deployed per health post to reduce workload and improving HEW incentives, including career development, may assist in increasing HEW job satisfaction, which in turn could help to reduce attrition, including leaving the health sector.

## Background

As a signatory of the Alma Ata Declaration “Health for All” and the Millennium Development Goals, the government of Ethiopia had the responsibility to significantly improve the health of the Ethiopian population. To achieve the goals set forth in those international commitments, the government conceived the idea of the Health Extension Program (HEP) in its second Health Sector Development Plan (HSDP II) with the aim of rapidly expanding health care services to inaccessible and underserved rural communities since 2003 [1]. Health posts at the lowest level of the tier system are the facilities where HEP services are provided in addition to services provided through household visits [2]. Services are provided by at least two female, college-trained and salaried health extension workers (HEWs) and are based on nationally developed packages tailored to the varying situations and requirements of the regions in the country [3]. The program was originally launched in 2003 in the country’s four agrarian regions (Amhara, Oromia, SNNPR and Tigray). Later, it was expanded to the pastoralist communities in 2006 and urban areas in 2010 [3]. The program was key in improving access to basic health services especially among agrarian and urban regions. A study by Tafesse et al. confirms this where health service utilization has been significantly improved among urban women as a result of the urban health extension program [4].

Healthcare workers are the greatest assets for a country’s health system. The presence of competent and motivated workers is crucial for proper running of health programs and meeting expected goals [5, 6]. Likewise, community health workers play an enormous role in bringing services closer to rural inhabitants. Community health programs could suffer from sustainability issues and high costs associated with recruitment and training of health workers if there is a high turnover [7]. Studies show that community health programs involving a large number of workers usually have a high rate of attrition due to various reasons, including lack of adequate support and supervision, poor recruitment process, absence of refresher training, insufficient pay, lack of family support, better opportunities in other fields and lack of recognition by the community, among others [7–10]. This is not an exception for the Ethiopian program if appropriate actions are not taken on the important factors that lead to increased attrition.

Before 2008, Ethiopia had a slow rate of urbanization. Economic activities and alternative job opportunities were limited [11, 12]. However, in the subsequent years since 2008, situations in the country have changed rapidly, including the availability of better employment there by increasing HEWs’ chances of leaving their job [12, 13]. The cost of living has also skyrocketed during the same period, with their salary not keeping the pace up [13]. Furthermore, the political volatility and increased violence across the country since 2015 may have had its own effect on the health sector, including HEW performance [14]. Such and other factors might test their will to remain motivated and to continue working as an HEW. The planned reform by the Ministry of Health to the existing program called the Second-Generation Health Extension Program barely has data to base the proposed changes on. Except for a few localized studies, there has not been a comprehensive nationwide study showing the magnitude, trend and regional variation of HEW attrition, which can act as a source of evidence to improve the program. Hence, this study was designed to describe the situation of HEWs since their deployment includes their status in terms of various indicators, including individual-level and institutional factors. The objectives of this study are estimating the magnitude and trend of attrition over the years, describing the HEWs whereabouts and jobs after leaving the program and determining the proportion of HEWs leaving the health sector. Understanding their status over the years, including the current status and the possible effects of the changing political and economic situations of countries on their attrition, would help to prepare for the challenges faced in the mission to achieve universal health coverage through primary healthcare.

## Methods and materials

### Study setting and period

Health Extension Worker’s personnel file of eight regions (Tigray, Afar, Amhara, Oromia, Benishangul Gumuz, SNNPR, Gambella and Harari) and two city administrations of Ethiopia (Addis Ababa and Dire Dawa city administrations) was reviewed from June—July 2019. The study was conducted in randomly selected district health offices and HEWs working within those districts. HEWs are accountable to their catchment Health Center and the District Health Office. According to the HEP guidelines, supervisors from the District Health Office and Health Centers provide various types of support and conduct regular supervision. Administrative services such as salary, promotion, leave and other benefits are provided in the District Health Office, and their personnel file is also located there [3]. Over 40,000 HEWs have also been trained and deployed in health posts since 2003, significantly contributing to an increase in the number of front-line healthcare workers, thereby becoming the backbone of increased rural health service coverage and utilization [15, 16]. At inception, the study was planned to be conducted in all nine regions and city administrations. However, data collection was not conducted in the Benishangul Gumuz and Somali regions due to security issues and poorly kept records of HEWs, respectively. This study assessed the attrition of HEWs from the initial implementation of the program in 2003 until the end of 2018.

### Study design

Mixed study designs involving both quantitative and qualitative methods were employed. A cross-sectional personnel records review was used to identify quantitative data on the sociodemographic, educational, administrative and other characteristics of HEWs and health posts. For the qualitative study, key informant interviews and focus group discussions were conducted on purposefully selected HEWs and health managers to assess pertinent reasons behind resigning from the Health Extension Program.

An existential-phenomenological qualitative research design was applied to HEWs who had left their positions (referred to as “leavers”) to understand the causes of attrition. Through this approach, we conducted an in depth, systematic understanding of the reasons behind the decision of HEWs to leave their job, including their values, purposes and intentions. Interviews were held with purposely chosen sample HEWs who recently left their position. Focus group discussions (FGDs) with health administrators employed at the regional and district levels were held to better understand managers' perspectives on the elements that contribute to HEW attrition.

### Study population

All health extension workers both actively working and those who already resigned from their position were the study population in this study. A representative sample of HEWs from selected districts was included in this particular study. Those HEWs who had been registered by the District Health Office Human Resources Department, including those who had resigned after deployment from their job for any reason, were considered eligible to be included in our study.

### Sample size determination and sampling procedure

One of the objectives of this study was to determine the proportion of attrition of the HEWs. Hence, the sample size was calculated using a single population proportion formula with a 5% level of significance, proportion of attrition of 7.2% among HEWs [8] and a 0.9% margin of error. Using this formula, the sample size including a 10% nonresponse rate was calculated to be 3,486.

**Fig. 1:**
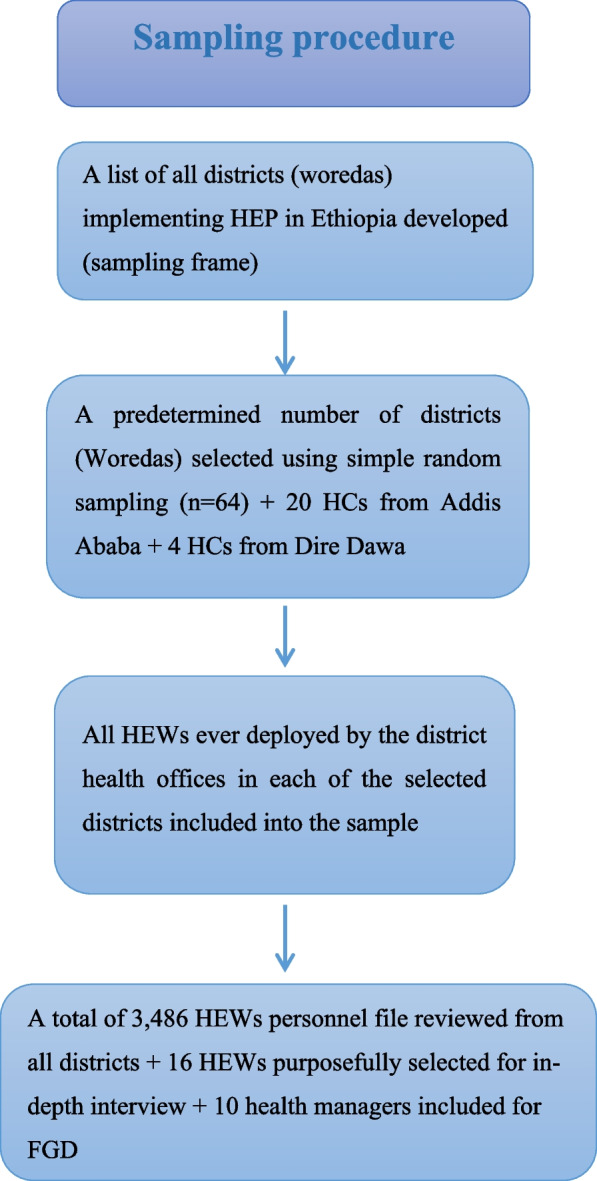
Flow chart showing the sampling procedure

In Ethiopia, district (woreda) health offices are responsible for employing and keeping records of health extension workers. In city administrations, health centers (HCs) take over this role. Initially, district health offices were randomly selected from among a list of districts implementing the Health Extension Program. Conducting the sampling together would lead to underrepresentation of the pastoralist and urban districts in the study, as the number of agrarian districts far outnumbers the other two. Hence, we conducted a separate sampling for the Agrarian, Pastoralist and Urban HEP implementation areas to ensure the proportional inclusion of districts of all types. In this way, a total of 64 district health offices and 24 health centers from Addis Ababa and Dire Dawa were sampled. Then, a cluster sampling method was employed to include the study participants until the needed sample size was reached, i.e., all health extension workers who had ever been deployed by the selected districts provided that their personnel file was found in the human resources records and contained the needed information. A focus group discussion with health leaders from the district and regional health bureau was held to explore reasons for resignation from the program, and a total of 16 HEWs who had already left their jobs were purposefully chosen for in-depth interviews (Fig. 1).

### Data collection and data quality control

Sources of data for review were human resources and financial documentation, such as hiring, transfer files and payroll documents located in the district health offices. To ensure the validity and reliability of the data collection tool, structured questionnaires were developed by the study team and were evaluated by experts consisting of biostatisticians, epidemiologists and other public health professionals before being pretested in one of the district health offices. Data collectors and supervisors were trained on the overall objectives of the study and the questionnaire. They were healthcare professionals with a minimum Bachelor’s degree. Daily call conferences were made between investigators and field supervisors, and immediate decisions were given for any errors occurring in the field. Data were collected using ODK on a tablet computer to ensure the quality and completeness of the entered data. Supervisors have made visits to the data collection sites and have assisted in reviewing the collected data on a daily basis together with the data collection team to resolve any inconsistencies to ensure that the intended questions have been answered properly. Collected data were sent to the central server daily via the internet. Feedback arising from the review of completed questionnaires was conveyed to the data collectors to improve the quality of the data collected. Qualitative data were collected using a semi-structured questionnaire initially prepared in English and then translated to local languages. Interviews were audio recorded using digital audio recorders, and additional field notes were taken alongside the audio recording by the data collectors.

### Data management and analysis

STATA version 14 (STATA Corporation, College Station, Texas 77,845 USA) and Microsoft Excel 2019 (Microsoft Corporation, WA, USA) were used for all statistical analyses in this study. The first outcome variable, attrition from HEP, was recoded into 0 (no) = continued working as an HEW and 1 (yes) = left job as an HEW. Left job as a HEW included resignation, disappearance without notification, death, official dismissal and change in qualification/position. The date of resignation or date removed from the payroll was used to estimate the attrition for each year. The other outcome variable is attrition out of the health sector based on their current job after leaving the HEP and has two categories, namely, “health sector job” and “non-health sector job”. Since the sampling was performed in two stages, i.e., first Districts and then HEWs, probability weight was assigned for each observation. Hence, all percentages in this article are weighted.

Descriptive analyses including measures of central tendency and variation, such as the mean, median, interquartile ranges, standard deviations, and proportions, were calculated to describe the study population. Frequency tables were produced to describe the demographic and other characteristics of the HEWs. A logistic regression model was used to estimate the magnitude and predictors of attrition of HEWs out of the health system, which is considered the worst form of attrition. Chi-square tests were performed to investigate the association between the outcome and predictor variables. A cutoff point of *p* <  = 0.25 was used to select variables for the multiple logistic regression model. Variables yielding a p value of less than 0.05 were considered statistically significant in the multivariable model. The Hosmer‒Lemeshow goodness-of-fit test and the ROC curve were used to assess model fitness.

Audio records of qualitative interviews were first transcribed into the local languages and then translated back to English for analysis. Interview notes and transcriptions of the audio files were combined. Line by line, segment by segment, and paragraph by paragraph coding was applied to the exported data. Following the completion of the coding system, all items were grouped into themes, and thematic analysis was used to describe the findings. Thematic content analysis was conducted using Nvivo version 12 software to identify the main themes that emerged from the data.

## Results

### Response rate of the study

A total of 88 districts (including Health Centers in Addis Ababa and Dire Dawa) were selected for inclusion. However, data were collected from 75 districts, and 13 districts, the Benishangul Gumuz and Somali regions, were not included. Most of the districts included were from Oromia [14], Amhara [10] and SNNPR [10], although the inclusion was made proportionally from all regions and city administrations. In total, 3,476 HEW personnel files were reviewed. In terms of the HEP implementation category, 2,505 (51.4%) of the HEWs were agrarian, 323 (11.3%) were pastoralist, and 648 (37.3%) were urban.

### Description of the sociodemographic characteristics of HEWs in Ethiopia

The results show that 3,391 (96%) HEWs were female, and most of them were in the age range of 20 to 24 years. Approximately 63.8% of the HEWs were born in rural areas. (Table 1). The average distance of the health posts from the district health office was 19 km (IQR 5 to 15). However, some health posts were more than 80 km away from WoHO (Woreda Health Office).

**Table 1:** Sociodemographic and other characteristics of HEWs in Ethiopia [*n* = 3,476], 2019

Variable	Category	Unweighted frequency	Weighted % [95%CI]
Sex	Male	85	4 [0.03–0.05]
	Female	3,391	96 [0.95–0.97]
Age category	18 to19	881	23.4 [0.21–0.25]
	20 to 24	1,954	56.4 [0.54–0.59]
	25 to 29	436	14.6 [0.13–0.16]
	30 and above	205	5.6 [0.05–0.07]
Birth place	Rural	2,444	64 [0.62–0.66]
	Urban	1,016	36 [0.34–0.38]
Marital status at deployment	Single	2,697	76.9 [0.75–0.79]
	Married	734	22.0 [0.20–0.24]
	Divorce/separated/widowed	29	1.1 [0.00–0.02]
Recent marital status	Married	1,611	56.3 [0.54–0.59]
	Still single	1,092	43.7 [0.41–0.46]
Number of children	No children	1,267	42.7 [0.40–0.45]
	One or more children	2,030	57.3 [0.55–0.60]
Disability	Yes	14	0.3 3 [0.001–0.005]
	No	3,446	99.7 [0.994–0.998]

### Career development, administrative issues and performance of HEWs

Regarding their education level, only 66 (3.9%) of them were below grade 10, while 3,399 (96.1%) of the HEWs had an education level of 10th grade and above. Furthermore, 159 (4.3%) had another certification in addition to the Health Extension Program. The results also show that 2,347 (67%) of the HEWs were COC certified. Only 69 (2.6%) of them had previous work experience before joining the HEP, and most of them had two or three years of experience.

With respect to their certification level at the time of deployment, approximately 2,535 (63.7%) had a level 3 certification, 463 (19.2%) had a diploma-level certification, and only 8 (0.3%) HEWs had a degree-level certification. Their most recent certification (i.e., after deployment) shows that 1,935 (46.9%) still had a level 3 certification, whereas 384 (16%) had a diploma-level certification and approximately 4.8% of the HEWs had a degree-level certification.

Nearly 60% of the HEWs had taken at least one annual leave during their career, and only 4% of the HEWs had at least one recognition. The two common reasons for recognition were best performance and innovative work. Recognitions were either in kind or in cash together with certification. On the other hand, 915 (23%) HEWs had some kind of administrative reprimand at some point during their career. Reasons included absence from work (88%), low performance (9%), and other unspecified reasons (3%). Reprimands included salary reduction, dismissal and legal actions.

The performance of HEWs is recorded in the personnel files of HEWs every year. It is measured out of 100% on different attributes, such as achievement of planned activities, work attendance and supervisor’s appraisal. The results showed that the most recent median performance of HEWs was 80% (IQR 76% – 86%). Approximately 63% of those who had left their job had a lower than the median performance.

### Regional and district level variation of attrition of HEWs

The overall attrition during the 15 years of implementation was 21.1%. The highest attrition was observed in Addis Ababa at 30.3%, followed by Amhara (18.2%) and Afar (12.1%). On the other hand, the lowest attrition rates were seen in Harari (3.2%), Dire Dawa (3.8%) and Gambella (4%). Among the HEWs who had left their job, 27.3% formally submitted a resignation letter, while 39.8% simply disappeared without noticing their employer, which was the district health office. Additionally, 2.9% of attrition was due to death, while 4.7% were dismissed from their job for various reasons, including absenteeism from work and poor performance. (Table 2).

**Table 2:** Frequency distribution of HEWs attrition in Ethiopia [*n* = 727], 2019

**Variable**	**Category**	**Unweighted frequency**	**Weighted % [95%CI]**
Attrition from HEP	No	2,749	78.9 [0.77–0.81]
	Yes	727	21.1 [0.19–0.23]
Attrition by region	Tigray	47	8.7 [0.065–0.114]
	Afar	22	36.1 [0.082–0.174]
	Amhara	193	25.3 [0.156–0.209]
	Oromia	131	9.2 [0.077–0.109]
	SNNPR	137	10.5 [0.087–0.125]
	Gambella	21	4.0 [0.026–0.061]
	Harari	3	3.2 [0.010–0.093]
	Addis Ababa	157	30.3 [0.264–0.346]
	Dire Dawa	16	3.8 [0.023–0.061]
Attrition by district type	Agrarian	463	45.6 [0.411–0.499]
	Pastoralist	88	17.1 [0.131–0.221]
	Urban	176	37.3 [0.328–0.421]
Type of attrition	Resignation	191	27.3 [0.236–0.314]
	Disappearance	304	39.8 [0.354–0.444]
	Death	23	2.9 [0.017–0.048]
	Dismissal	49	4.7 [0.033–0.067]
	Transfer	43	6.6 [0.041–0.104]
	Change in position	117	18.7 [0.152–0.228]

### Trend of attrition of HEWs

**Fig. 2:**
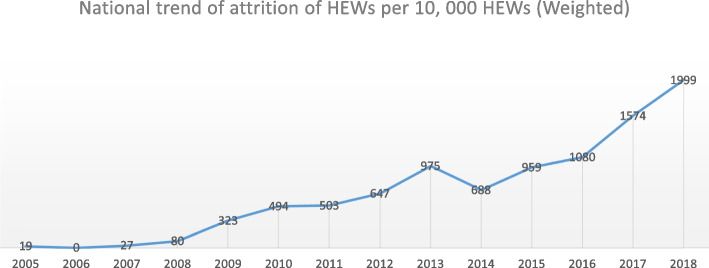
Trend of HEW attrition in Ethiopia from 2005 – 2018 [per10, 000]

The national trend shows that the earliest attrition occurred right the next year after the initial launch of the program in 2005 and had a magnitude of 19 per 10,000 HEWs. In the subsequent years after 2006, the trend of attrition continued to steadily increase except for a slight decrease in 2014 to 688 per 10,000 HEWs. The year 2018 saw an all-time high attrition of 1,999 HEWs per 10,000. (Fig. 2).

### Magnitude and predictors of attrition of HEWs from the health sector and their whereabouts

A total of 704 HEWs had their job status recorded after leaving HEP. Overall, 502 (71.3%) of the HEWs left the health sector, while 202 (28.7%) left their HEP position but continued to work in the healthcare sector. In the bivariate analysis, variables such as age, sex, marital status at and after deployment, previous work experience, number of languages spoken, legal penalty and annual leave had a *p* value > 0.25 and were not considered further.

Model diagnostics including goodness of fit and multicollinearity tests were performed for the multiple logistic regression model. Accordingly, the model was found to be a good fit as the p-value for the Hosmer Lemeshow goodness was 0.9947. The mean VIF for all the variables was 1.13 indicating absence of correlation among the predictor variables. Additionally, the tolerance values for each of the variables in the final model ranged from 0.76–0.97 again confirming the absence of multicollinearity. Initially, place of birth, school grade level, certification level both at deployment and during resignation and having special skills became insignificant predictors. However, the number of biological children, having an additional education, receiving official recognition, administrative reprimand, distance between district health office and health post and COC status were found to have a statistically significant association with leaving the health sector after resignation.

HEWs having one or more children had 39% lower odds of leaving the health sector after resignation compared to those having no children (AOR = 0.61, 95% CI; 0.42–0.89) after controlling for the other variables. HEWs with no additional education were 8.3 times more likely to leave the health sector than those who had additional education (AOR = 8.3, 95% CI; 3.67–18.98). Being recognized at least once was associated with 71% lower odds of leaving the health sector after quitting working as an HEW (AOR = 0.29, 95% CI; 0.10–0.83). (Table 3).

**Table 3:** Bivariate and multivariate analysis results of factors associated with attrition out of the health sector [*n* = 478], 2019

**Variables**		**Attrition out of the Health Sector **		**COR (95% CI)**	AOR (95%CI) *significant at ***p*<0.05**
		**No (%)**	**Yes (%)**		
Place of birth	Urban	103 (35.4)	188 (64.6)	1	1
	Rural	94 (23.5)	306 (76.5)	1.78 (1.27–2.49)	0.89 (0.54–1.47)
Number of children	None	69 (24.7)	201 (75.3)	1	1
	One or more	120 (31.5)	261 (68.5)	0.71 (0.50–1.01)	0.61 (0.42–0.89) *
Elementary school grade	Below grade 10	2 (11.8)	15 (88.2)	1	1
	Grade 10 and above	198 (29.1)	482 (70.9)	0.32 (0.07–0.26)	0.76 (0.16–3.46)
Additional education	Yes	25 (69.4)	11 (30.6)	1	1
	No	172 (26.3)	483 (73.7)	0.16 (0.09–1.44)	8.34 (3.67–18.98) *
Certification level during deployment	Level one	9 (18)	41 (82)	1	1
	Level two	2 (11.7)	15 (88.3)	1.6 (0.32–8.51)	0.74 (0.05–10.35)
	Level three	100 (22.5)	344 (77.5)	0.76 (0.35–1.61)	0.61 (0.42–6.11)
	Level four	13 (44.8)	16 (55.2)	0.27 (0.09–0.76)	2.22 (0.43–11.50)
	Diploma nurse	76 (47.2)	85 (52.8)	0.25 (0.11–0.54)	2.22 (0.29–17.22)
	Degree (N/MW)	2 (66.7)	1 (33.3)	0.11 (0.01–1.34)	17.1 (0.57–513.7)
Possessing special skills	Yes	25 (69.4)	11 (30.6)	1	1
	No	172 (26.3)	483 (73.7)	5.12 (1.52–17.21)	1.27 (0.31–5.30)
Official recognition	No	183 (27.3)	488 (72.7)	1	1
	Yes	14 (70)	6 (30)	0.16 (0.06–0.42)	0.29 (0.10–0.83) *
**Variables**		**Attrition out of the Health Sector**		**COR (** **95% CI)**	**AOR (95%CI)**
		**No (%)**	**Yes (%)**		
Certification level during resignation	Level one	6 (14)	37 (86)	1	1
	Level two	1 (6.7)	14 (93.3)	2.27 (0.25–20.58)	2.34 (0.25–22.31)
	Level three	75 (19.5)	310 (80.5)	0.67 (0.27–1.64)	0.84 (0.33–2.15)
	Level four	28 (38.4)	45 (61.6)	0.26 (0.09–0.69)	0.44 (0.15–1.28)
	Diploma nurse	33 (28.5)	83 (71.5)	0.41 (0.16–1.06)	0.61 (0.21–1.77)
	Degree (N/MW)	54 (91.5)	5 (8.5)	0.02 (0.01–0.05)	0.02 (0.01–0.09)
Administrative reprimand	No	151 (33)	306 (67)	1	1
	Yes	46 (19.7))	188 (80.3)	2.02 (1.38–2.94)	1.66 (1.07_2.56) *
Distance from district office to Health Post	1–15 km	136 (35.3)	249 (64.7)	1	1
	15 km or more	61 (20)	245 (80)	2.19 (1.54–3.11)	1.75 (1.18–2.59) *
COC status	CoC certified	121 (39.5)	185 (60.5)	1	1
	CoC not certified	76 (19.7)	309 (80.3)	2.66 (1.89–3.74)	2.06(1.39–3.06) *

On the other hand, being reprimanded administratively was associated with a 66% higher likelihood of leaving the health sector (AOR = 1.66, 95% CI; 1.07 -2. 56). HEWs working in health posts with a distance of 15 km or more from the district health office were 75% more likely to leave the health sector (AOR = 1.75, 95% CI; 1.18–2.59). HEWs having no COC certificate were nearly twice as likely to leave the health sector compared to those who were COC certified (AOC = 2.06, 95% CI; 1.39–3.06). (Table 3).

Regarding the HEWs whereabouts after leaving the health sector, most of them were reported to be residing either in the district where they used to work (42%) or in the region (29.6%). However, approximately 4.3% of them reportedly went abroad mostly to the Gulf countries in search of jobs.

### Reasons for attrition: qualitative findings

Four key themes emerged from the qualitative analysis as reasons for attrition of HEWs: (1) psychosocial; (2) administrative/structural; (3) salary and incentive-related; and (4) working environment.

Under the psychological factors category, a variety of issues affected the HEW attrition rate. One category of factors falls under the heading of personal factors. Inadequate skills, personal beliefs about their work, personal conflicts, health-related issues, a lack of community trust and respect for HEWs, and/or a lack of community value for HEP and/or HEWs are a few examples. One HEW described her experience as follows.

*“…. We are from rural communities; we think that we would get into the town when recruited. However, the reality is not like that; we work with the rural community after the training. Therefore, since we work in rural areas, we develop an attitude that we have not improved. However, whenever we think that we are working to benefit society, we won’t think to leave the job for a second. However, as I mostly observed most of us want to live and work in urban areas.”* IDI, Amhara region

Under the administrative factors category, several issues affecting HEW attrition have been identified, such as unresponsive district health officers, officers' negative perception, poor support practices, unequal treatment or bias, disrespect of officials, demanding false reports, judgmental appraisals, denying legitimate leave, denial of educational opportunities, and officials who break their promises. One participant from a focus group discussion said…

*“Because they stay there for a long time, they develop burnout syndrome. I believe that this is one of the reasons that makes them leave their job. Moreover, motivations, including salary increments, incentives from mobile and transportation and other issues, are not afforded by district health offices due to budget shortages. Therefore, there is nothing to keep them motivated, which might lead to more attrition. The other observation regarding reasons for attrition is personal health problems. When they develop chronic disease, they are more likely to leave their jo...”* <Files\\Attrition FGD with RHB, WoHO.>

Salary- and incentive-related factors such as low pay, a low chance of promotion, benefits, and incentive programs have been identified as reasons facilitating leaving their job. The following is what was said by an employee of an RHB.

*“On my side, what I would like to add is that all the new packages and activities that have been added are additional workloads to the HEWs. All the responsibilities of other sectors down at the kebele level, such as education, agriculture and women’s affairs, have been shifted to HEWs. Although some packages have been added, nothing has been considered for HEWs. There is no top up or increment in their salary. Therefore, they receive the same salary for all additional activities. While doing all those activities, they earn the same salary as teachers. When the packages were only 16 in number, there were no problems, but now there are so many complaints”* Files\\Attrition FGD with RHB, WoHO

Work environment factors were the other theme that emerged from the qualitative analysis. Distance to the work site, available transportation options, and challenging topography have all been covered under the category of work environment as factors enhancing attrition of the HEWS. A HEW described the difficulty of topography and long-distance travel as follows.

*“People gather at the church and that is where health education is given. However, the place is mountainous. Therefore, it is difficult to climb the mountain and go there to give education, and it is also very far. There is also another place where the water overflows during the winter. Therefore, we go there by crossing the water body. In addition, the name of the place we were assigned is called ‘Zuria’, and it is considered close to the place where we live. However, the villages are very far apart. After giving vaccination in one village, we have to cross the water body or travel a long distance to reach another village.”* <Files\\KII_Attri-MO3_Amhara_Semen Mecha>

## Discussion

Our study has shown that there is a variation in the rate of attrition among regions in the country ranging from 6.1% to 38.5%, with the national average being 21.1%. While there is no internationally agreed definitive cutoff point to designate a certain level of attrition as low, medium or high, the figure in this study could be considered relatively low considering the long period for which attrition was measured, which is 15 years. However, for a country that almost entirely relies on these workers to deliver the crucially needed basic preventive and curative health services communities at the periphery, the increasing trend of attrition is concerning.

Usually, the magnitude of attrition is weighed in terms of the value and performance of those employees who leave their job. Since the method of measuring the performance of HEWs nationally is not well standardized, it might be difficult to accurately classify HEWs as high or low performers. However, such measures could enable us to define a healthy level of attrition rate by simply identifying the least performers, as the leaving of such employees would pave the way for new talent and energy to achieve goals. The current study has analyzed data collected on the performance of HEWs as documented by district health offices. It shows that since such measurements began, the majority (63%) of those who scored below the average performance of 80% have left the job. In this sense, it could be justified that such attrition where mostly the low performers leave is advantageous. It is also good to note that some of the attritions are due to official dismissal because of frequent absenteeism, discipline issues and related factors, which again justifies that such attritions are beneficial for the program. Death also contributed to approximately 3% of the attrition in this study, which is an unavoidable cause.

Among the regions, the earliest attrition occurred in the Amhara region in 2005, with a magnitude of 19 per 10,000 HEWs, and has since occurred every year. The second earliest attrition occurred in the SNNPR and Dire Dawa city administration in 2007, with magnitudes of 7.8 and 24 per 10,000 HEWs, respectively. These three regions showed the longest continuous streak of attrition among all the regions in the country. In Tigray, attrition was first observed in 2010 with a magnitude of 151 per 10,000 HEWs, the most delayed among the regions that initially launched HEP. The shortest streak of attrition was seen in the Harari and Gambella regions starting in 2017 and 2013, respectively, with magnitudes of 20 and 109 per 10,000 HEWs, respectively.

Various studies have reported the attrition rate of community health workers in different countries. Community health extension workers and community health officers are front-line healthcare workers formally trained, employed and salaried in Nigeria. A study on attrition in a Nigerian state revealed that approximately 53.3% of all attrition in the state was due to these community health workers, which is higher than that of other professionals, such as nurses and midwives [17]. Although the study is not exclusively on the attrition of community health workers, it indicates a higher level of attrition among such workers. Brazil is also another country that integrates community health agents into civil services and hence is recognized as a professional and receives a salary. Although they have contributed greatly to the improvement of various national indicators, the presence of high turnover is the greatest challenge [18]. However, another study in South Africa focused on tuberculosis treatment has seen an attrition of 11 out of 12 lay health workers in the community within a one-year period. This is an overwhelmingly high attrition, although the sample size is very small to comfortably compare the results of the study to the current one [19].

A study in Bangladesh conducted to assess the factors affecting the recruitment and retention of community health workers in community-based newborn care identified a 74% (32 out of 43 workers) attrition rate over a four-year period. However, the study included a small sample size, which might not have adequate power to accurately estimate the attrition rate [20]. An unpublished study by Aberra Feyissa [21] found a 27% attrition rate of HEWs in the Oromia region of Ethiopia, which is slightly higher than the current national figure. However, the current study found the attrition rate to be 9.2% in the Oromia region, far lower than the previous study. Contrary to the above findings and the current study, a study conducted in Nepal on female community health workers in a program that lasted for over 20 years has seen an attrition of less than 5% annually. Similar to the current finding, variations were seen among different districts of the country where it was higher than the average in some of the districts [22]. A seven-year prevalence of attrition of 49.6% is seen in a Kenyan study on community health workers, showing a higher attrition rate compared to the current study [7]. Namibia is one of the countries implementing the Health Extension Program, similar to Ethiopia. In an evaluation of the overall program, it was found that there was a low level of attrition annually at only 3.6%. However, attrition varied considerably among the regions in the country [23].

The attrition rate of HEWs has varied over the years just as it did among the regions during the 15 years of implementation of the program. The first five years until 2008 saw the lowest magnitudes of attrition, and attrition was absent in 2006. However, since 2009, it has continually increased until 2018, with the highest magnitude of 1,999 per 10,000 HEWs. The low magnitude during the early years of implementation is expected and could be attributed to a few job opportunities in other sectors, the majority of HEWs not being married and the fact that the program itself was initiated in the rural parts of the country. As the years went by, HEP implementation was further expanded into urban and pastoralist areas, many HEWs who were single become married, other job opportunities started to grow and in most regions for most of the implementation period incentives such as annual leave, transfer and educational opportunities were restricted all of which might have contributed to the increasing attrition in the later years. The higher trend of attrition also tended to coincide with the overall political instability in the country, especially from 2016 to 2018.

Similar studies conducted in other countries also show variation in attrition during different years. A study in Uganda shows a retention rate of 95% during the first year and 91% and 86% during the second and 5^th^ years of implementation, respectively, an increasing trend. The study concludes that retention of the majority of community health workers over a medium term is possible [24]. In a study in Kenya on a community-based program started in 2002, while the overall attrition was 33%, there was a drop out of 36% in the first two years. In 2007, five years after the initiation of the program, 59% of the staff had dropped out, showing an enormous increase in attrition with a similar trend to the current study. The study points out high expectations, and the subsequent rejection by beneficiary families was the main reason for the high attrition during the later years [6]. In another related study on the retention of community health workers in Tanzania, an attrition rate of 12.7% over a four-year period was observed. The trend of attrition of community health workers varied over the years. In the first year, 174 community health workers remained, followed by 123 and 174 community health workers in the second and third years, respectively [25].

Oftentimes, HEWs who leave their job due to promotion to district health offices are tasked with supervising HEWs. Others occasionally join local NGOs working in the area and are usually engaged in a related field of work in the health sector. Despite their turnover, their continued work in the health sector is beneficial, as their years of expertise are utilized. However, the results in our study show that the majority of HEWs leaving their job also quit working in the health sector altogether. This could be considered the worst form of attrition considering the investment made on them during their initial training as well as while on the job.

One of the limitations of this study is the fact that some regions were not included in the study for various reasons, including the absence of the needed data and security issues. Because of this, the overall attrition may be slightly underestimated. The trend of attrition could also look different, as these are pastoralist regions with peculiar challenges causing increased attrition of HEWs from the onset of the program.

## Conclusion

Our finding reveals a relatively low magnitude of attrition of HEWs but a high variation in the rate of attrition among regions. There is also evidence that out-of-health sector attrition is very high among the leavers, indicating a loss of important expertise that could have contributed greatly to the health sector. Having an additional education, being administratively reprimanded and having a long distance between the health post and district health office all increased attrition out of the health sector, while having children, official recognition and COC certification tended to reduce out-of-health sector attrition. Nonetheless, the program is able to retain a large proportion of them given the duration of the program, which was in its 15th year in 2018. In-depth studies should be conducted to identify the attributes of workers leaving their job, as it is preferable to retain better performers.

## Data Availability

The raw dataset of this study can be obtained from the corresponding author upon reasonable request.

## Abbreviations

ETB: Ethiopian Birr
IQR: Interquartile Range
IRB: Institutional Review Board
HEP: Health Extension Program
HEWs: Health Extension Workers
HSDP: Health Sector Development Program
SNNPR: Southern Nations Nationalities and Peoples Region
